# Dynamic Service Function Chain Deployment and Readjustment Method Based on Deep Reinforcement Learning

**DOI:** 10.3390/s23063054

**Published:** 2023-03-12

**Authors:** Jing Ran, Wenkai Wang, Hefei Hu

**Affiliations:** 1 School of Electronic Engineering, Beijing University of Posts and Telecommunications, Beijing 100876, China; ranjing@bupt.edu.cn; 2 School of Information and Communication Engineering, Beijing University of Posts and Telecommunications, Beijing 100876, China; huhefei@bupt.edu.cn

**Keywords:** network function virtualization, service function chain, dynamic deployment, resource allocation, deep reinforcement learning, deep Q-networks, network readjustment

## Abstract

With the advent of Software Defined Network (SDN) and Network Functions Virtualization (NFV), network operators can offer Service Function Chain (SFC) flexibly to accommodate the diverse network function (NF) requirements of their users. However, deploying SFCs efficiently on the underlying network in response to dynamic SFC requests poses significant challenges and complexities. This paper proposes a dynamic SFC deployment and readjustment method based on deep Q network (DQN) and M Shortest Path Algorithm (MQDR) to address this problem. We develop a model of the dynamic deployment and readjustment of the SFC problem on the basis of the NFV/SFC network to maximize the request acceptance rate. We transform the problem into a Markov Decision Process (MDP) and further apply Reinforcement Learning (RL) to achieve this goal. In our proposed method (MQDR), we employ two agents that dynamically deploy and readjust SFCs collaboratively to enhance the service request acceptance rate. We reduce the action space for dynamic deployment by applying the M Shortest Path Algorithm (MSPA) and decrease the action space for readjustment from two dimensions to one. By reducing the action space, we decrease the training difficulty and improve the actual training effect of our proposed algorithm. The simulation experiments show that MDQR improves the request acceptance rate by approximately 25% compared with the original DQN algorithm and 9.3% compared with the Load Balancing Shortest Path (LBSP) algorithm.

## 1. Introduction

Network operators often face significant challenges in delivering services efficiently. Traditional network architectures rely heavily on specialized hardware devices known as middleboxes to provide various network functions (NFs). However, this approach can result in high capital and operating expenses. To address this issue, Network Function Virtualization (NFV) unbundles specific network functions (e.g., firewalls, Deep Packet Inspection) from hardware and allows them to run on general-purpose devices (e.g., X86 servers) [1]. Moreover, Software Defined Networking (SDN) technology enables flexible deployment and traffic scheduling of NFV by decoupling the control plane from the data plane [2]. Service Function Chain (SFC) consists of a specific sequence of Virtual Network Functions (VNFs), each of which can be deployed on a virtual machine of the underlying network and flexibly scaled or migrated to other servers [3,4].

In domains such as 5G networks, cloud computing, and data centers, we need various services to meet the user demands. Therefore, it is worthwhile to study how to quickly and flexibly deploy SFCs on the underlying network according to users’ needs [5]. This deployment process requires optimal utilization of computing resources while also satisfying latency and bandwidth constraints and arranges the deployment location of SFC/VNF reasonably to provide superior services to users. This problem belongs to the class of NP-hard problems [6], which are at least as hard as any problem in NP. NP stands for nondeterministic polynomial time, which denotes the class of problems that can be solved by a nondeterministic algorithm in polynomial time. NP-hard problems are known to be highly challenging and computationally expensive to solve efficiently. The deployment problem of SFC is divided into static deployment and dynamic deployment. In static deployment, all SFC requests are known before deployment, and an overall optimal solution for all SFC requests is required.

Dynamic deployment differs from static deployment in that SFC requests arrive dynamically, and an optimal deployment solution for each arriving SFC must be found on the basis of the current situation. Most existing traditional methods transform the SFC deployment problem into integer linear programming (ILP) or its transformed form [6,7,8,9]. However, these methods have high computational complexity and face difficulties in finding optimal solutions in large-scale networks. To reduce the complexity of ILP-based methods, some studies have proposed heuristic algorithms [10,11,12]. However, these algorithms rely on the assumption that the network can be well-modelled and predicted. Therefore, they need to be redesigned when the network conditions change [13], which makes them unsuitable for dynamic deployment. Recently, Machine Learning approaches have also been applied to the problem of resource provisioning in VNF/SDN networks [14,15]. In Reinforcement Learning (RL), agents learn strategies for achieving specific goals by maximizing returns during interactions with the environment. RL has proven its strength in solving combinatorial optimization problems [16]. However, given that the dynamic SFC deployment problem has a large set of states and actions, the classical RL algorithm, Q-learning, needs to maintain a vast Q-table and suffers from low algorithmic performance due to limited computational resources. Deep reinforcement learning (DRL) has gained increasing popularity, as it combines RL with deep learning (DL) to overcome the limitations of traditional reinforcement learning methods. DQN uses neural networks to replace Q-tables, increasing the speed of calculation, and is now also used in the dynamic SFC deployment problem [17,18,19,20,21,22,23]. Most existing studies on dynamic SFC deployment, however, overlook the need for the readjustment of deployed SFCs, which is a meaningful aspect of the problem [24,25,26].

In this paper, the objective is to deploy SFCs dynamically in an SDN/NFV-enabled network, aiming to achieve higher request acceptance rates. The deployment needs to consider the bandwidth and CPU resource requirements of SFC requests while ensuring their end-to-end latency requirements. The main contributions of this paper are as follows:

1. We propose MQDR, a DRL-based framework for dynamic deployment and readjustment of SFCs. This framework employs two trained agents, $A^{*}$ and $B^{*}$, to select VNF deployment locations by $A^{*}$ and adjust the underlying network by $B^{*}$, respectively, to enable SFC requests to be received successfully when such requests arrive dynamically. We first present the use of these two agents together to address the dynamic SFC deployment problem.
2. We impose restrictions on the action space to simplify the training of the agents and enhance the deployment performance. Unlike allowing the agents to determine deployment locations among all nodes, MQDR incorporates the M Shortest Path algorithm (MSPA) to reduce the range of actions to be chosen by the agents.
3. Finally, we compare MQDR with other methods, and it is found that MDQR improves the request acceptance rate by approximately 25% compared with the original DQN algorithm and by 9.3% compared with the Load Balancing Shortest Path (LBSP) algorithm. Therefore, the proposed method is a feasible solution to the dynamic SFC deployment problem.

The remainder of this paper is organized as follows: Section 2 provides a summary of previous related work conducted by other researchers; Section 3 presents the network architecture, the system model, and the formulation description of the research problem; Section 4 describes our MQDR algorithm in detail; Section 5 shows simulation experiments and performance evaluation; and Section 6 concludes and discusses future work.

## 2. Related Works

Traditional network function equipment can lead to a long update cycle of network service and high operating expenses. In response to the practical need to solve these problems, SDN and NFV have become research focuses in the relevant field in recent years [27,28,29]. On the basis of SDN/NFV technologies, SFC defines a sequence of ordered VNFs. In earlier studies, the SFC deployment problem was usually modelled as an optimization problem and solved by integer linear programming (ILP) or its deformation. Zhong et al. [7] proposed an ILP model to minimize the cost of deploying SFCs among data centers. Bari et al. [6] considered minimizing operational costs while maximizing resource utilization and proposed a corresponding ILP model. Savi et al. [8] considered the impact of network function location on deployment costs and proposed an ILP model. Addis et al. [9] developed an MILP model to minimize the CPU resources used to instantiate the VNF. However, most of these studies focused on the static deployment of SFCs and did not consider that SFC requests are constantly arriving.

Compared with ILP, heuristic algorithms can reduce the calculation time to obtain sub-optimal solutions. Rankothge et al. [10] proposed an SFC resource allocation framework using genetic algorithms. The ILP takes several hours to compute for SFC deployment in a network of 16 servers, while their heuristic algorithm takes only a few milliseconds. Jin et al. [11] proposed a depth-first search algorithm to select paths and a path-based greedy algorithm to allocate VNFs when applying the MILP problem to a large network scenario. Wu et al. [12] proposed a heuristic algorithm to jointly optimize end-to-end latency, resource consumption, and network load balancing on the basis of SRv6. Although heuristic algorithms can deploy SFCs sequentially, they may lack flexibility and are unsuitable for their dynamic deployment of SFCs. Additionally, these algorithms are susceptible to quickly falling into local optima, which limits their effectiveness.

Machine Learning (ML) has emerged as a powerful tool for various domains in recent years [30,31,32,33]. Some studies have leveraged it to tackle the challenges of SFC deployment problem. Tang et al. [14] solved the node overload problem by predicting the resources required for SFCs on the basis of a deep belief network prediction algorithm. For nodes that are predicted to be overloaded, each VNF on them is migrated to the underlying node that satisfies the resource threshold constraint by a greedy algorithm based on merit selection. Subramanya et al. [15] considered a neural network to predict traffic, followed by deployment with heuristic algorithms. Among the available studies, deep learning was used mainly for traffic prediction, aiding other deployment methods.

In Reinforcement Learning (RL), the agent struggles to obtain the strategies that maximize returns during interaction with the environment. RL has been applied to various research areas, including complex networking and communications problems [17]. Several studies have recently applied RL to the SFC deployment problem. Sun et al. [18] proposed a method that combines graph neural networks with RL for SFC deployment. Li et al. [19] proposed an adaptive SFC deployment method that chooses between two heuristic algorithms using DQN. However, choosing between two heuristic algorithms leads to a limited range of actions. Wang et al. [20] utilized RL to determine to which data center the SFCs and standby SFCs are deployed. The authors proposed five backup-level schemes to improve fault tolerance. However, this study considered only where SFCs are deployed and not where VNFs are deployed. Gu et al. [21] proposed an Intelligent VNF Orchestration and Flow Scheduling model via Deep Deterministic Policy Gradient (DDPG), aided by a heuristic algorithm to accelerate the training progress. However, the model in that paper assumes that the underlying resources are unlimited and that traffic requests can be predicted in advance, which is inconsistent with reality. Pei et al. [22] formulated a Binary Integer Programming (BIP) model and obtained the placement of VNFs by Double Deep Q-network (DDQN). When resources were insufficient, they considered horizontal scaling, which initiated new instances. However, this study also assumed that traffic can be predicted in advance. Qiu et al. [23] proposed a DQN-based online SFC deployment method. The algorithm minimizes the total resource consumption overhead while satisfying the request delay constraint and improves the request acceptance rate of the operator’s network. However, this algorithm does not limit the action space of the DQN, which poses a challenge for training the algorithm effectively in practice.

There already exists some research on the readjustment of SFCs. Fu et al. [24] applied DQN to adapt SFCs to changing traffic in IoT networks and proposed decomposing VNFs into components, but did not elaborate on the implementation details. Tang et al. [25] proposed a traffic prediction method and designed two dynamic VNF instance scaling algorithms. Liu et al. [26], proposed a Dyna-Q-based SFC readjustment algorithm to balance the load of the underlying network and demonstrated its advantages over the baseline algorithm in terms of overhead and running time. However, these studies focused mainly on adjusting existing SFCs to adapt to traffic changes and did not address how to accommodate new SFC requests.

Compared with existing studies, we consider constraints on the action space when applying DRL and consider network readjustment for newly arrived SFC requests. These changes have paid off and improved the request acceptance rate.

## 3. System Model and Problem Description

This section provides a detailed description of the VNF/SFC-based network model, followed by a formulation of the dynamic deployment and readjustment problems of SFCs. The problem is then defined as a Markov decision process (MDP).

### 3.1. Network Model

**Figure 1:**
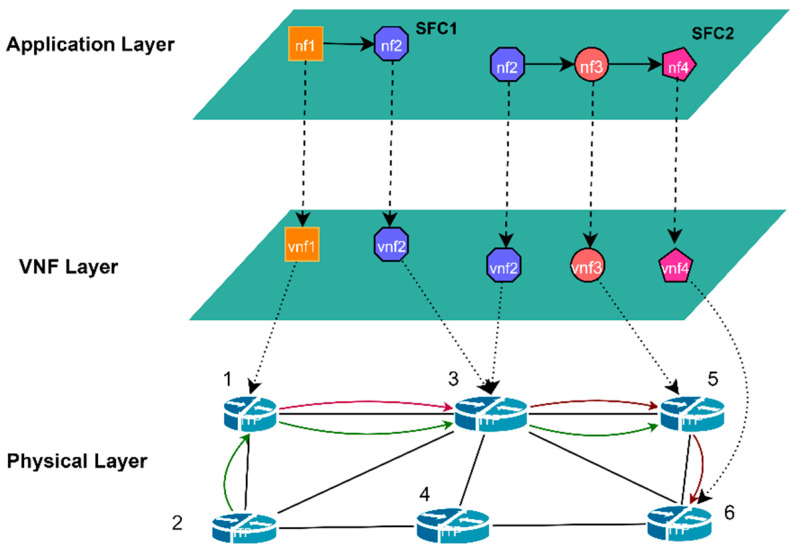
Three-layer NFV network architecture.

The network architecture considered in this study is a three-layer NFV architecture [34,35], as depicted in Figure 1 (the Notations used in the model are shown in Table 1). The physical layer consists of physical nodes (servers) connected by physical links in the underlying network. In the VNF layer, VNFs are instantiated on physical nodes that provide the required resources. In the application layer, different NFs are deployed on the corresponding type of VNFs to form SFCs.

The underlying network is modeled as a fully connected undirected graph, denoted as $G=(V^{S},E^{S})$, where the set of nodes is represented by $V^{S}$, and the set of links is represented by $E^{S}$. Each underlying node in the network has a CPU resource capacity denoted as $C_{i}^{S}$. The physical link between node $v_{{}_{i}}^{s}$ and node $v_{{}_{j}}^{s}$ is represented by $e_{ij}^{s}\in E^{S}$, with a bandwidth capacity of $BW_{ij}^{S}$ and a latency of $L_{ij}^{S}$.

### 3.2. SFC and VNF

The set of VNF types is denoted as F, where each VNF has its own type f. $coeff_{c}^{f}$ represents the CPU resource factor, indicating the amount of CPU resources consumed per unit of traffic passing through the VNF of type f. $\theta^{f}$ represents the traffic scaling factor, indicating the multiple by which traffic passing through the VNF is of type f. The set of SFC requests is denoted by R. Each request $r^{j}\in R$ can be denoted as $r^{j}=[v_{j,in}^{s},v_{j,out}^{s},V_{j}^{NF},E_{j}^{NF},l_{j},z_{j}{}^{s},z_{j}{}^{e}]$, where the ingress is $v_{j,in}^{s}$, and the egress is $v_{j,out}^{s}$. $V_{j}^{NF}$ denotes the ordered set of NF requests, which is the set of VNF types through which $r^{j}$ passes in order. The CPU resource requirement of the u-th NF $v_{j,u}^{nf}\in V_{j}^{NF}$ is $c_{j,u}^{nf}$.

The traffic segment of $r^{j}$ is denoted as logical link $e_{j,uv}^{nf}\in E_{j}^{NF}$, where $bw_{j,uv}^{nf}$ represents its bandwidth requirement, while $l_{j}$ represents the maximum end-to-end delay allowed for the request. We assume that the traffic requested by the SFC does not change after arrival, and $\left[z_{j}{}^{s},z_{j}{}^{e}\right]$ represents the service period of $r^{j}$.

For each SFC request $r^{j}$, we use a Boolean variable $p_{j}$ to show whether it has been successfully deployed (the Notations of Decision variables are shown in Table 2).

The variable $t_{j,u}^{f}\in \{0,1\}$ specifies the type of NFs in the SFC. It is noteworthy that input and output nodes do not deploy VNFs, i.e., $t_{j,u}^{f}$ equals 0.

(1) $$t_{j,u}^{f}=\left\{\begin{array}{c} 1,\\ 0, \end{array}\right.\begin{array}{c} \;v_{j,u}^{nf}\mathrm{is}\;\mathrm{type}\;\mathrm{of}\;f\in F\\ \mathrm{else} \end{array}$$

The decision variable $\eta_{j,u}^{i,t}\in \{0,1\}$ is introduced to show whether the *u*-th virtual node $v_{j,u}^{nf}\in V_{j}^{NF}$ of $r^{j}$ is deployed on physical node $v_{i}^{s}\in V^{S}$ during t. We partition time into discrete time slots, with each slot representing a specific temporal interval. The variable t is introduced to represent one of these time slots.

(2) $$\eta_{j,u}^{i,t}=\left\{\begin{array}{c} 1,\\ 0, \end{array}\right.\begin{array}{c} \;v_{j,u}^{nf}\;\mathrm{is}\;\mathrm{deployed}\;\mathrm{on}\;v_{i}^{s}\;\mathrm{during}\;t\\ \mathrm{else} \end{array}$$

The decision variable $\tau_{j,uv}^{pq}\in \{0,1\}$ is introduced to show whether the traffic segment $e_{j,uv}^{nf}\in E_{j}^{NF}$ of $r^{j}$ flows through the physical link $e_{pq}^{s}\in E^{S}$ during t.

(3) $$\tau_{j,uv}^{pq,t}=\left\{\begin{array}{c} 1,\\ 0, \end{array}\right.\begin{array}{c} e_{j,uv}^{nf}\;\mathrm{deployed}\;\mathrm{on}\;e_{pq}^{s}\;\mathrm{during}\;t\;\\ \mathrm{else} \end{array}$$

If an NF of type f maps to $v_{i}^{s}\in V^{S}$, then $v_{i}^{s}$ must also instantiate a VNF of type f. The decision variable $\beta_{f}^{i}\in \{0,1\}$ is introduced to show whether there is a VNF of type f on physical node $v_{i}^{s}\in V^{S}$.

(4) $$\beta_{f}^{i}=\left\{\begin{array}{c} 1,\\ 0, \end{array}\right.\begin{array}{c} \mathrm{there}\;\mathrm{is}\;a\;\mathrm{VNF}\;\mathrm{of}\;\mathrm{type}\;f\;\mathrm{on}\;v_{i}^{s}\\ \mathrm{else} \end{array}$$

(5) $$if\;\beta_{f}^{i,t}=1,\;\sum_{r^{j}\in R}\sum_{v_{j,u}^{nf}\in V_{j}^{NF}}\eta_{j,u}^{i,t}t_{j,u}^{f}\geq 1,\;\forall v_{i}^{s}\in V^{S},\;f\in F,\;t\in T$$

The CPU resource requirements of $v_{j,v}^{nf}\in V_{j}^{NF}$ can be determined by the incoming traffic bandwidth:

(6) $$c_{j,v}^{nf}=bw_{j,uv}^{nf}\sum_{f\in F}t_{j,v}^{f}coeff_{c}^{f}$$

The traffic may experience scaling as it passes through each VNF, resulting in changes to the required bandwidth:

(7) $$bw_{j,uv}^{nf}=bw_{j,ku}^{nf}\sum_{f\in F}t_{j,u}^{f}\theta^{f}$$

$v_{k}^{nf},v_{u}^{nf},v_{v}^{nf}\in V_{i}^{NF}$ is a set of sequential NFs.

We define the readjustment of SFC as an NF migration process. For instance, the migration of the u-th NF of $r^{j}$ from $v_{i}^{s}$ to $v_{i^{\prime }}^{s}$ can be defined as follows:

(8) $$\left\{\begin{array}{l} \eta_{j,u}^{i,t}=1\Rightarrow \eta_{j,u}^{i,t}=0\\ \eta_{j,u}^{i^{\prime },t}=0\Rightarrow \eta_{j,u}^{i^{\prime },t}=1 \end{array}\right.$$

Simultaneously, the mapping between the logical links $e_{j,ku}^{nf}$ and $e_{j,uv}^{nf}$ of $r^{j}$ and the corresponding physical links undergoes a change. Let $E^{s_{1}}$ be the set of physical links passing through the original deployment route and $E^{s_{2}}$ be the set of physical links passing through the post-migration deployment route. Thus, we can express this change as:

(9) $$\left\{\begin{array}{l} \tau_{j,uv}^{pq,t}=1\Rightarrow \tau_{j,uv}^{pq,t}=0,\forall e_{pq}^{s}\in E^{s_{1}}\\ \tau_{j,uv}^{p^{\prime }q^{\prime },t}=0\Rightarrow \tau_{j,uv}^{p^{\prime }q^{\prime },t}=1,\forall e_{p^{\prime }q^{\prime }}^{s}\in E^{s_{2}} \end{array}\right.$$

The above variables satisfy the following constraints: each VNF of the SFC must be deployed on a single physical node, and each virtual link must be mapped onto a single physical link:

(10) $$\begin{array}{cc} \sum_{v_{i}^{s}\in V^{S}}\eta_{j,u}^{i,t}=1 & \forall r^{j}\in R,\;\forall v_{j,u}^{nf}\in V_{j}^{NF},\forall t\in T \end{array}$$

(11) $$\begin{array}{cc} \sum_{e_{pq}^{s}\in E^{S}}\tau_{j,uv}^{pq,t}=1 & \forall r^{j}\in R,\;\forall e_{j,uv}^{nf}\in E_{j}^{NF},\forall t\in T \end{array}\;$$

The CPU resources available on the node must satisfy the following constraint:

(12) $$\begin{array}{cc} \sum_{r^{j}\in R}\sum_{v_{j,u}^{nf}\in V_{j}^{NF}}\eta_{j,u}^{i,t}c_{j,u}^{nf}\leq C_{i}^{S} & \forall v_{i}^{s}\in V^{S},\forall t\in T \end{array}$$

The underlying link bandwidth must satisfy the following constraint:

(13) $$\begin{array}{cc} \sum_{sfc^{j}\in S}\sum_{e_{j,uv}^{nf}\in E_{j}^{NF}}\tau_{j,uv}^{pq,t}bw_{j,uv}^{nf}\leq BW_{pq}^{S} & \forall e_{pq}^{s}\in E^{S},\forall t\in T \end{array}$$

SFC must satisfy the following latency constraint:

(14) $$\begin{array}{cc} \sum_{e_{j,uv}^{nf}\in E_{j}^{NF}}\sum_{e_{pq}^{s}\in E^{S}}\tau_{j,uv}^{pq,t}L_{pq}^{S}\leq l_{j} & \forall r^{j}\in R \end{array}$$

Every connected pair of VNFs must satisfy the principle of traffic conservation:

(15) $$\begin{array}{l} \sum_{v_{q}^{s}\in \Omega^{+}(v_{p}^{s})}\tau_{j,uv}^{qp,t}-\sum_{v_{q}^{s}\in \Omega^{-}(v_{p}^{s})}\tau_{j,uv}^{pq,t}\\ =\left\{\begin{array}{c} \eta_{j,v}^{p,t}-\eta_{j,u}^{p,t},\\ 1,\\ -1,\\ 0, \end{array}\right.\begin{array}{c} \forall v_{j,u}^{nf},v_{j,v}^{nf}\in V_{j}^{NF}\\ \forall v_{j,v}^{nf}\in \{v_{j,out}^{s}\},\;v_{j,out}^{s}=v_{p}^{s},\;v_{j,u}^{nf}\in V_{j}^{NF}\\ \forall v_{j,u}^{nf}\in \{v_{j,in}^{s}\},\;v_{j,in}^{s}=v_{p}^{s},\;v_{j,v}^{nf}\in V_{j}^{NF}\\ \mathrm{else} \end{array},\forall r^{j}\in R,\forall t\in T \end{array}$$

$\Omega^{+}(v_{p}^{s})$ and $\Omega^{-}(v_{p}^{s})$ represent the set of upstream and downstream nodes, respectively.

Our objective is to deploy as many SFC as possible, which can be represented by maximizing the SFC request acceptance rate:

(16) $$\max\frac{\sum_{r^{j}\in R}p_{j}}{\left|R\right|}$$

### 3.3. Markov Decision Process (MDP)

The dynamic deployment and readjustment of SFC is a complex and multi-faceted process that can be approached using RL, which is based on the MDP. RL is employed to identify the optimal policy, which is a mapping of states to actions aimed at maximizing the final cumulative return. 

There are two main categories of methods used in DRL: (1) value-based methods, such as Deep Q Network (DQN), and (2) policy-based methods, such as Policy Gradient (PG). The difference between them is as follows [36]: (1) Value-based methods are more appropriate for action spaces that are discrete and low-dimensional, while policy-based methods are better suited for continuous action spaces. (2) The value-based methods provide the value of actions, whereas the strategy-based methods provide the most valuable action directly. (3) The value-based methods update the agent after each step, whereas the strategy-based methods update them after each episode.

We then formally define dynamic SFC deployment and readjustment as a Markov decision process. Typically, a Markov decision model is defined as 〈S,A,P,R,γ〉, where S represents the set of states, A represents the set of discrete actions, and P:S×A×S represents the probability distribution function of state transfers. R:S×A represents the reward function. γ∈[0,1] represents the discount factor of the current reward value to the future—the higher the discount factor, the more attentive the agent is to the impact of the current step on the future.

State: We define each state $s_{i}\in S$ as a vector $s_{t}=[C_{t},BW_{t},I_{t}]$, where $C_{t}=[C_{t}{}^{1},C_{t}{}^{2},\ldots ,C_{t}{}^{\left|V^{s}\right|}]$ represents the CPU resource of the underlying network, and $BW_{t}=[BW_{t}{}^{1},BW_{t}{}^{2},\ldots ,BW_{t}{}^{\left|E^{s}\right|}]$ represents the available bandwidth resource of the underlying physical link. $I_{t}=[r^{j},v_{j}{}^{l},l_{j}^{t}]$ represents the state of the currently deployed SFC, $r^{j}$ represents the currently arriving SFC request, $v_{j}^{l}$ represents the last NF deployed physical node, and $l_{j}^{t}$ denotes the sum of the latency of all used paths of the SFC.

In DRL, an agent selects an optimal action on the basis of the current state S. In the deployment and readjustment, we define the actions chosen by the agent separately as follows:

Deployment Action: Let $k=1,2,\ldots ,|V^{s}|$ be the indexes of the nodes in the network. An action a∈A is an integer where $A=\{0,1,2,\ldots ,|V^{s}|\}$. If a=0, no physical nodes are available for deployment. Otherwise, a indicates the node index where the NF is deployed.

Readjustment Action: Let $k=1,2,\ldots ,|V^{s}|$ be the indexes of the nodes in the network. An action a∈A is an integer where $A=\{0,1,2,\ldots ,|V^{s}|\}$. If a=0, there are no physical nodes to be adjusted. Otherwise, a indicates the node index where we carry out the readjustment.

Reward: Our objective function is to maximize the service request acceptance rate. Therefore, the reward function is defined as:

(17) $$\left\{\begin{array}{l} 1,\mathrm{when}\;\mathrm{sfc}\;\mathrm{is}\;\mathrm{deployed}\\ 0,\mathrm{else} \end{array}\right.$$

State Transition: The state transition of MDP is defined as $\left(s_{t},a_{t},r_{t},s_{t+1}\right)$, where $s_{t}$ denotes the current state, $a_{t}$ is the action taken by the agent in the current state, $r_{t}$ is the reward received for taking $a_{t}$, and $s_{t+1}$ is the resulting state.

This section provides a detailed description of the VNF/SFC-based network model, which serves as the basis for formulating the dynamic problem of SFC deployment and readjustment. The problem is then framed as a Markov Decision Process, enabling the application of DRL to address it.

## 4. Algorithm Design

This section presents the framework of the DQN-based dynamic SFC deployment and readjustment algorithm, which uses trained RL agents to make decisions directly. Subsequently, a detailed description of the DQN-based dynamic SFC deployment and readjustment algorithm and its training process is provided.

### 4.1. DQN-Based Dynamic SFC Deployment and Readjustment Framework (MQDR)

**Figure 2:**
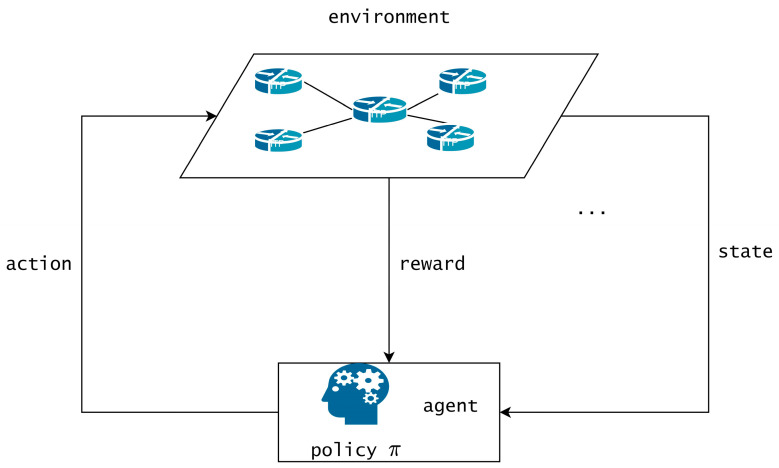
Reinforcement Learning.

In the previous section, we adopted the MDP to continually and automatically describe the transition of actions and states. Reinforcement learning can find the optimal policy given a Markov decision process, where the policy maps states to actions. As illustrated in Figure 2, the agent interacts with the environment by perceiving the current state and selecting an action from the available set of actions. Following the execution of the chosen action, the agent receives a reward signal from the environment, and the system transitions to the next state. The agent leverages these transitions to learn from the environment during the training phase.

**Figure 3:**
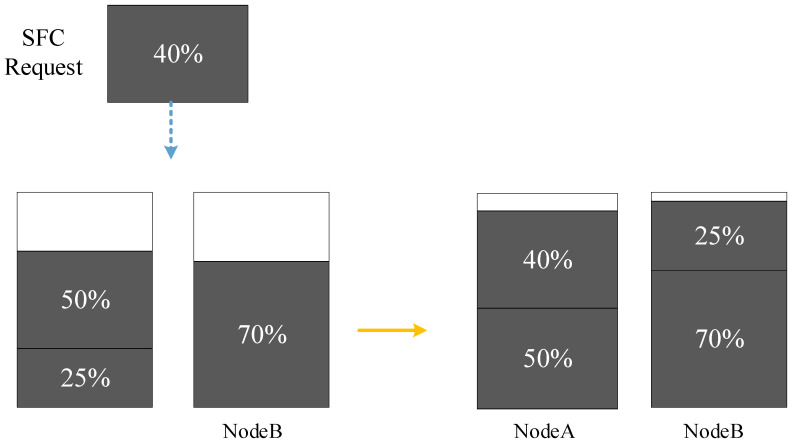
SFC readjustment.

Most existing studies on the dynamic SFC deployment problem neglect the readjustment of the deployed SFCs, which can be beneficial in some cases. Figure 3 shows an example of such a case, where NodeA and NodeB have equal CPU resources, and the grey squares represent the CPU resources occupied by the VNFs. Suppose NodeA’s CPU resources are 75% occupied and NodeB’s CPU resources are 70% occupied. A new SFC request that requires 40% of the CPU resources cannot be deployed successfully. However, if we move a VNF that occupies 25% of CPU resources from NodeA to NodeB, we can deploy the new SFC request after the readjustment [37]. Moreover, the readjustment enables us to deploy the new SFC on a path with lower latency to meet the requested latency requirements.

**Figure 4:**
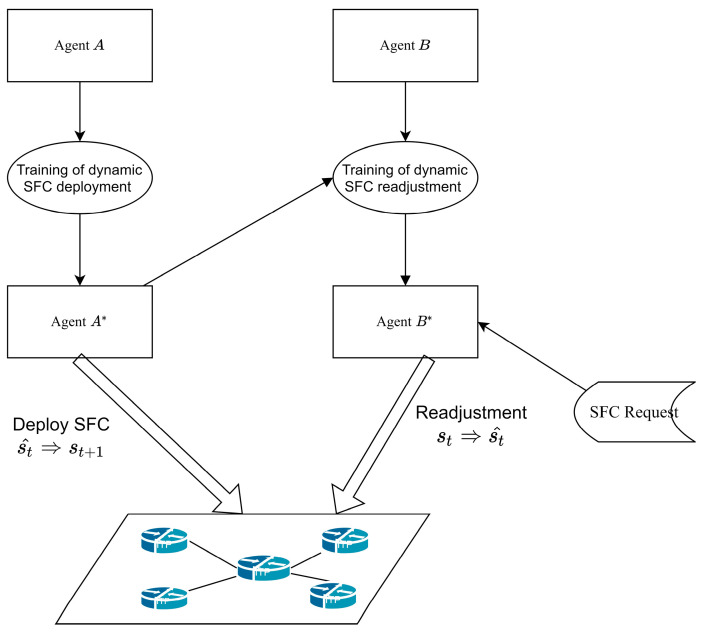
The framework of MQDR.

We propose a dynamic SFC deployment and readjustment method based on a deep Q network and M Shortest Path Algorithm (MQDR), which is illustrated in Figure 4. When a request arrives dynamically, it is first readjusted for the underlying network by the well-trained agent $B^{*}$. Agent $B^{*}$ adjusts some of the serving SFCs in the underlying network on the basis of the current network conditions and the arriving SFC request. It migrates some VNFs on the nodes that are close to resource saturation and creates conditions for the arriving SFC to be deployed. Following the adjustment, agent $A^{*}$ is responsible for the dynamic deployment of the SFCs. The two agents work collaboratively towards the goal of maximizing the number of SFC requests that can be received.

As discussed in the previous section, the action space for both SFC dynamic deployment and dynamic readjustment is discrete. Therefore, we utilized the DQN algorithm, which is a value-based algorithm that supports discrete actions. DQN combines neural networks and Q-learning. It inputs the current state directly to the neural network, computes the value of all actions and outputs the best one.

Figure 4 depicts our framework which involves two agents, $A^{*}$ and $B^{*}$. In multi-agent reinforcement learning, the following relationships may exist among agents: (1) cooperative relationship, where multiple agents have the same goal as each other; (2) competitive relationship, where one agent’s gain is another agent’s loss; (3) mixed relationship, where agents cooperate with some and compete with others; and (4) egoistic relationship, where agents care only about their own gain. Since both A and B aim to deploy more SFCs, they have a cooperative relationship. However, due to the added complexity of multi-agent reinforcement learning, directly applying the same training method as single-agent reinforcement learning may result in inadequate performance. For instance, if we train A and B simultaneously and B finds an optimal policy at some point while A does not, then B’s policy may change in the next iteration due to A’s influence. Consequently, B’s original optimal policy may no longer be optimal. This mutual interference may prevent the agents from converging to stable strategies. To avoid this problem, we propose obtaining the optimal deployment policy for A first and then training the readjustment policy for B without changing A.

In this study, the neural network parameters of Agent A depicted in Figure 4 are generated via a process of random initialization. Subsequently, the neural network parameters are trained through dynamic deployment in the training environment to obtain an optimal policy denoted by $A^{*}$. Next, we randomly initialize the neural network parameters of agent B and perform readjustment training. In each step of this process, agent B first readjusts its underlying network and then deploys the SFC by following agent $A^{*}$’s policy. This leads to agent $B^{*}$, which has the optimal readjustment policy.

The neural network of agents consists of an input layer, an output layer, and three fully connected layers for processing intrinsic information. The input to this neural network is the current environmental state vector, and the output is the Q-value of each action. As analyzed in the previous section, the input state $s_{t}=[C_{t},BW_{t},I_{t}]$, where $\left|C_{t}\right|=\left|V^{s}\right|$, $\left|BW_{t}\right|=\left|E^{s}\right|$, $\left|I_{t}\right|=9$, and the number of neural nodes in the input layer is $\left|V^{s}\right|+\left|E^{s}\right|+9$. The output corresponds to the index of a node chosen from the current network for deployment or readjustment operations, so the number of output nodes is $\left|V^{s}\right|$.

### 4.2. DQN-Based Dynamic SFC Deployment Algorithm

This section presents the DQN-based dynamic SFC deployment algorithm and its training process. Algorithm 1 shows the pseudocode of our algorithm. It takes as input the initial network state and the set of SFC requests $r_{1},r_{2}\cdot \cdot \cdot r_{m}$. After training, it outputs the dynamic deployment policy $\Pi_{1}$ (agent $A^{*}$).

The action-value function Q and the target action-value function $\hat{Q}$ are initialized, and $\hat{Q}$ is reset to Q every C steps. In the following C steps, we use $\hat{Q}$ to generate the target value y and use it to update Q. With the assistance of $\hat{Q}$, we can enhance the overall stability of the training process [38].

During the training, the environment is reset and a new set of SFC requests is initialized in every episode. It is worth noting that the agent is not informed of the SFCs in advance, only once they arrive.

For DQN, the more actions that can be selected, the more difficult it is to train. We, therefore, consider some rules to narrow down the selectable actions. Firstly, nodes that fail to satisfy resource and delay conditions are excluded. Secondly, we prioritize shorter paths to minimize latency and link resource usage. Nodes are sorted on the basis of the delay from the last Network Function (NF) deployment location (or input node), and the nearest m nodes are selected as the set Φ. Φ represents the truly selectable action space for agent A. The benefits of this approach are demonstrated in our experiments.

At each step, we select actions by ε−greedy to increase the “exploration” space of the agent. The complete state transition $e_{t}=(s_{t},a_{t},r_{t},s_{t+1})$ during this step is subsequently stored in the experience replay pool. During the training process, we sample a small batch from the experience replay pool and use the $\hat{Q}$ to generate a target $y_{j}$, which is the sum of the current reward and the discounted future reward. The neural network parameters θ are then updated using the gradient descent method.
**Algorithm 1:** DQN-based dynamic SFC deployment algorithm
**Input:** The underlying network state $s_{t}$ the set of dynamically arriving SFC requests $r_{1},r_{2}\cdot \cdot \cdot r_{m}$. **Output:** Dynamic SFC deployment policy $\Pi_{1}$. 1: Initialize the action-value function $Q(s_{t},a;\theta )$ where θ are the randomly generated neural network weights. 2: Initialize the target action-value function $\hat{Q}(s_{t},a;\theta^{-})$, where $\theta^{-}=\theta$. 3: Initialize the experience pool D with memory N. 4: **for** episode in range (EPISODES): 5:  Generate a new collection of SFCs. 6:  Initialize state s…. 7:  **for** step in range (STEPS):    8:     Select the nodes that satisfy the resource and delay requirements.  9:     Select m nodes that are closest to the last deployed node among the nodes that satisfy the deployment requirements and add them to set Φ.   10:    With probability ε, select an action $a_{t}$ at random.   11:    Otherwise, select the action $a_{t}=\arg\max_{a}Q(s_{t},a;\theta ),\;a\in \Phi$.  12:    Execute action $a_{t}$ and observe reward $r_{t}$.   13:    Store transition $e_{t}=(s_{t},a_{t},r_{t},s_{t+1})$ in D.  14:    Sample random minibatch of transitions $\left(s_{j},a_{j},r_{j},s_{j+1}\right)$ from D.  15:    Set $y_{j}=\left\{\begin{array}{l} r_{j},r_{j}=end\\ r_{j}+\gamma \max_{a^{\prime }}\hat{Q}(s_{j+1},a^{\prime };\theta^{\prime }),r_{j}\neq end \end{array}\right.$  16:    Perform a gradient descent step on ${\left(y_{j}-Q(s_{j+1},a;\theta )\right)}^{2}$ with respect to the network parameters θ.  17:    Every C steps, reset $\hat{Q}=Q$.  18:  End.  19: End.

### 4.3. DQN-Based Algorithm for Dynamic SFC Readjustment

This section presents the DQN-based dynamic SFC readjustment algorithm and its training process. Algorithm 2 takes as input the network state, SFC requests, and the dynamic SFC deployment policy $\Pi_{1}$ (agent $A^{*}$) and outputs the dynamic readjustment policy. The basic training framework of Algorithm 2 is similar to that of Algorithm 1, which also uses target network, experience replay, and gradient descent methods.

At each step, the agent identifies nodes that need readjustment due to excessive CPU resource usage and adds them to the set Φ. Then, the agent selects a node from those that need readjustment. The target node is selected by (1) filtering out nodes with sufficient resources and (2) selecting the nearest one as the target node. Compared with the way the agent selects both the source and target nodes for readjustment, the action space is reduced from ${\left|V^{S}\right|}^{2}$ to $\left|V^{S}\right|$, which significantly simplifies the neural network training.
**Algorithm 2:** DQN-based dynamic SFC readjustment algorithm
**Input:** The network state $s_{t}$ the set of SFC $G_{1},G_{2}\cdot \cdot \cdot G_{m}$, dynamic SFC deployment policy $\Pi_{1}$. **Output:** Dynamic SFC readjustment policy $\Pi_{2}$. 1: Initialize the action-value function $Q(s_{t},a;\theta )$, where θ is the randomly generated neural network weights. 2: Initialize the target action-value function $\hat{Q}(s_{t},a;\theta^{-})$, where $\theta^{-}=\theta$. 3: Initialize the experience pool D with memory N. 4: **for** episode in range (EPISODES): 5:  Generate a new collection of SFCs. 6:  Initialization state s. 7:  **for** step in range (STEPS): 8:     Generate the set of nodes that need to be readjusted based on the state of the underlying network. 9:     With probability ε, select an action $a_{t}$ at random. 10:    Otherwise, select the action $a_{t}=\arg\max_{a}Q(s_{t},a;\theta )$. 11:    Execute readjustment action $a_{t}$, $s_{t}\Rightarrow s_{t}{}^{\prime }$. 12:    Perform deployment with $\Pi_{1}$. 13:    Observe reward $r_{t}$$,\;s_{t}{}^{\prime }\Rightarrow s_{t+1}$. 14:    Store transition $e_{t}=(s_{t},a_{t},r_{t},s_{t+1})$ in D. 15:    Sample random minibatch of transitions $\left(s_{j},a_{j},r_{j},s_{j+1}\right)$ from D. 16:    Set $y_{j}=\left\{\begin{array}{l} r_{j},r_{j}=end\\ r_{j}+\gamma \max_{a^{\prime }}\hat{Q}(s_{j+1},a^{\prime };\theta^{\prime }),r_{j}\neq end \end{array}\right.$ 17:    Perform a gradient descent step on ${\left(y_{j}-Q(s_{j+1},a;\theta )\right)}^{2}$ with respect to the network parameters θ. 18:    Every C steps, reset $\hat{Q}=Q$. 19:  End. 20: End.

Once the readjustment node is selected, the agent moves the NF with the highest resource share on that node to the target node. Readjustment can improve the load balancing of the network and increase its capacity to accommodate more SFCs. After readjustment, the SFC is deployed by the dynamic deployment policy $\Pi_{1}$.

### 4.4. Conclusions

This section introduces the framework of the DQN-based approach to dynamic SFC deployment and readjustment (MQDR), as well as the specific details of its two main components. MQDR differs from other existing research work in two aspects: (1) it integrates dynamic readjustment of deployed SFCs with the deployment of newly arrived SFC requests, which can improve request acceptance rates, whereas existing research has addressed only either readjustment or deployment; and (2) it reduces the training difficulty and increase the practical value. The performance evaluation will demonstrate the benefits of these two features in detail.

## 5. Performance Evaluation

### 5.1. Simulation Setup

The algorithm proposed in this paper is evaluated on the CERNET2 network topology [39] using SFCSim [40], a Python-based SFC simulation platform. CERNET2 is the next-generation backbone network of the China Education and Research Network, which connects universities, research institutions, and other educational organizations across China. SFCSim employs a discrete-time event scheduling engine that supports the simulation of scenarios such as static and dynamic deployment of SFCs, service migration, and mobility management. The platform also implements various benchmark algorithms for experimental comparisons.

**Figure 5:**
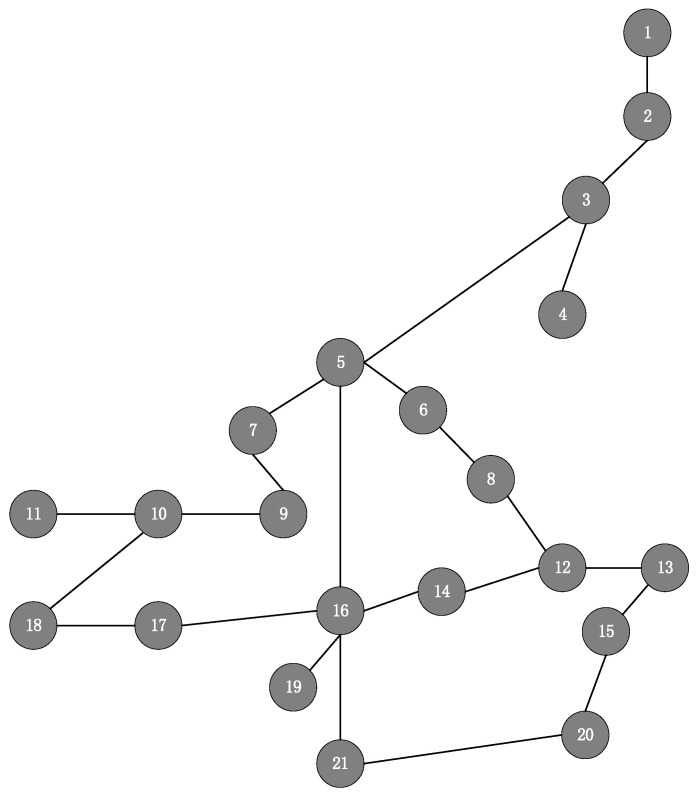
CERNET2 network topology.

The underlying network CERNET2 is depicted in Figure 5, with 21 physical nodes and 23 physical links, which can be abstracted as an undirected connectivity graph with 21 nodes and 23 edges. The server CPU resources are randomly generated within $\left[10,30\right]$ (units), the link bandwidth capacity to 20 Mbps, and the transmission delay is randomly generated within $\left[0.7,1.5\right]$ (ms). The SFCs arrive at different times, with their arrival time and service time following a uniform distribution in $\left[1200,10000\right]$ (units) and $\left[1000,1800\right]$ (units), respectively. Each SFC request contains 3~5 NFs, and its maximum SFC delay follows a uniform distribution in $\left[10,18\right]$ (ms). The traffic of each SFC is randomly generated within [0.1,0.6] (Mbps).

The experiments in this paper are conducted on a PC with an Intel i7-10750H CPU (6 cores and 12 threads), an Nvidia GTX2060 GPU (6 GB of video memory) and 16 GB RAM. The PC runs Windows 10 operating system and uses PyCharm IDE for simulation. The SFCSim simulation platform is integrated with TensorFlow 2.8 library based on Python 3.9 and tf-agents 0.12 library for the DQN simulation. The learning rate is set to 0.0005, the neural network parameters are learned by Adam [41] optimization, and each hidden layer has 512 nodes.

### 5.2. Experimental Results

**Figure 6:**
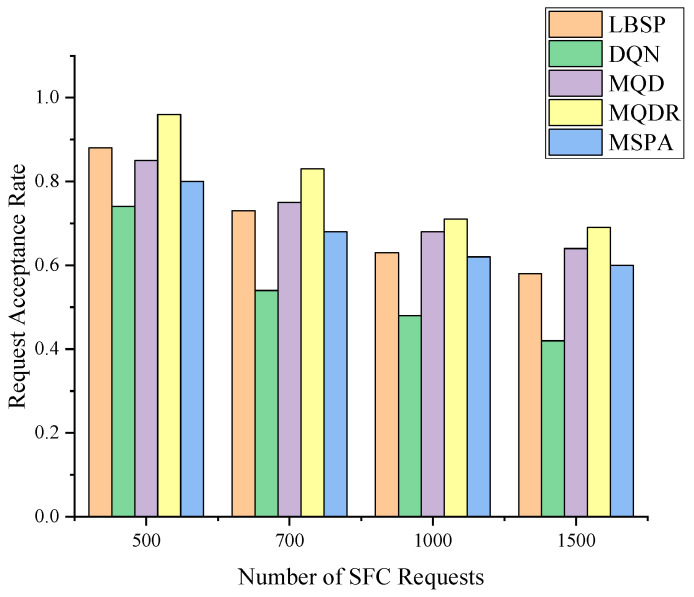
The request acceptance rate of different algorithms.

Figure 6 compares the request acceptance rates of the five different methods at different request quantities. We conducted 20 experiments for each request quantity and generated different sets of SFC requests according to the same parameters every time. We then obtained the average result. The five methods were as follows: (1) LBSP, a load-balanced shortest path algorithm [40], which deployed SFCs with fewer resources and used the shortest path delay route for stable performance; (2) MSPA, a method that selected the set of m nearest well-resourced nodes to the previous VNF, denoted as $\Phi_{m}$, and then randomly chose a node from $\Phi_{m}$ to deploy the next NF; (3) DQN, an online SFC deployment method based on deep Q networks [23]; (4) MQD combined DQN with MSPA, where the agent selected one node from $\Phi_{m}$ at each step; and (5) MQDR, which extended MQD by dynamically readjusting already deployed SFCs, as described in Section 4.

**Figure 7:**
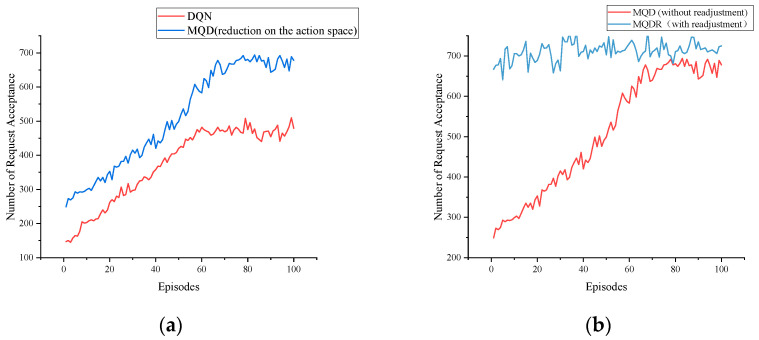
The training process comparison of DQN, MQN, and MQDR. (**a**) Compares the training performance of DQN and MQD, which reduces the action space based on DQN. (**b**) Compares the training performance of the MQD and the MQDR, where the MQDR adds readjustments to the MQD. MQD is the training base for MQDR.

**Figure 8:**
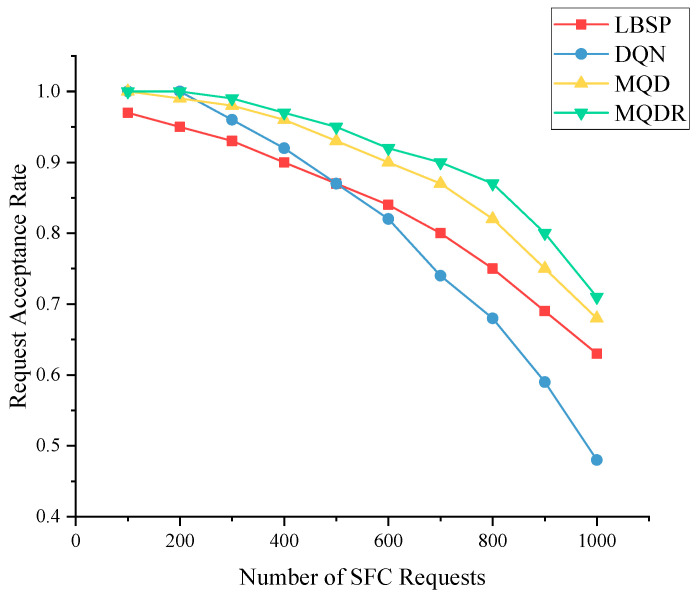
Performance of different algorithms with different number of requests.

Figure 7a,b show the training process of DQN, MQD and MQDR 3–5 when the SFC request quantity is 1000, and Figure 8 depicts the request acceptance rate of each algorithm as the requests arrive.

In the experiments, we adopted the ε−greedy method in DQN, MQD and MQDR and reduced ε from 0.9 to 0.05. The discount factor γ was set to 0.9. The parameter m of MSPA was set to 5 in MQD and MQDR.

As depicted in Figure 6, the request acceptance rate for all five algorithms decreased to different degrees as the quantity of SFC requests increased. As more underlying resources were occupied, subsequent SFC requests were less likely to be received and deployed successfully. LBSP outperformed MSPA at low to medium volumes, whereas they had similar performance at high volumes. This indicates that LBSP was a superior method, and MSPA introduced some uncertainty by randomly selecting from $\Phi_{m}$, which did not achieve better results than SPA.

MQD was the second most effective method overall. It improved the request acceptance rate by an average of 5.5% compared with MSPA by using DQN instead of randomly selecting actions from $\Phi_{m}$, and it outperformed LBSP. When comparing MQD with DQN, as depicted in Figure 7a, MQD also achieved a significant improvement over DQN by limiting the action space, which saved bandwidth resources and reduced the difficulty of training compared with selecting a node from all nodes.

MQDR outperformed MQD and achieved the best performance among all algorithms. This is because MQDR dynamically adjusted the existing deployment on the basis of the current underlying status when each new request arrived, which led to a more balanced allocation of resources and reduced the negative factors that hindered the deployment of new requests.

**Figure 9:**
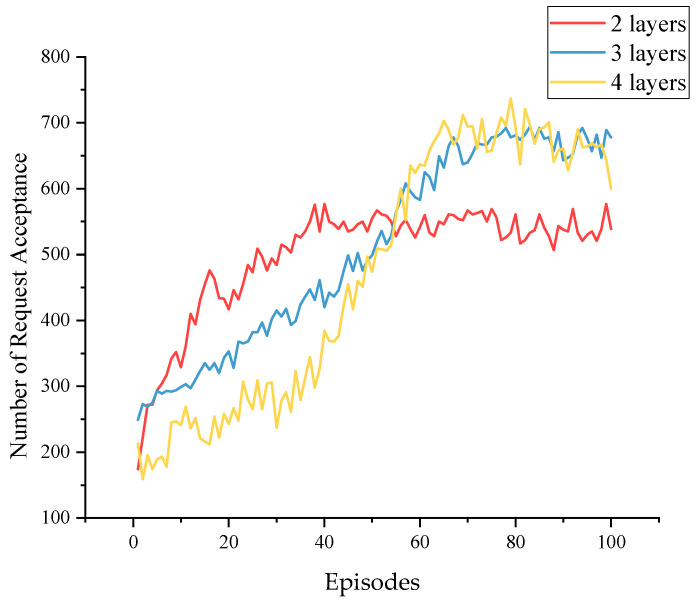
The training process varies with the number of hidden layers.

Figure 9 shows the Q-network’s different training processes with different hidden layers. Table 3 presents their performance in the test environment. The figure indicates that the three-layer and four-layer networks achieved similar results in training, while the three-layer network performed better in the test. Too many layers may cause overfitting and gradient instability during training. The two-layer networks performed the worst, as they were not deep enough to perceive all the intrinsic features.

**Figure 10:**
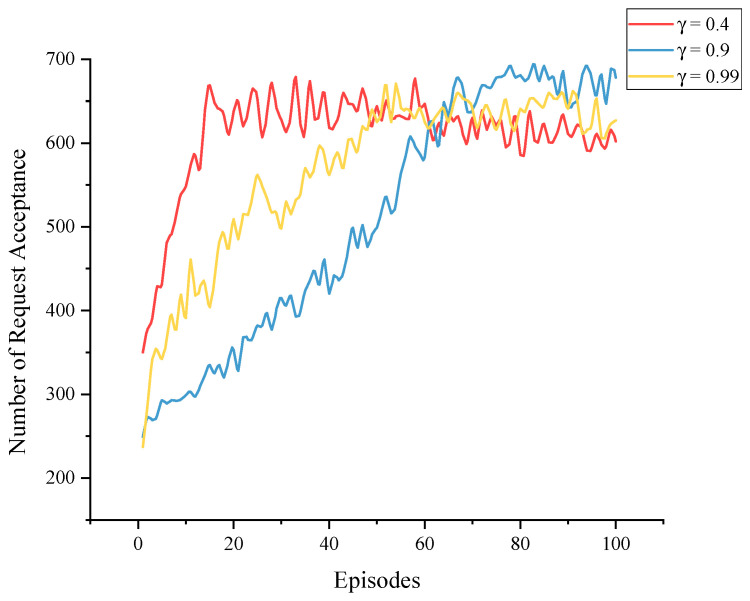
Training processes with different γ values.

Figure 10 shows the effect of different discount factors γ on the training of the agents. The discount factor γ is used to calculate the target value during training and to regulate the immediate and long-term effects in reinforcement learning, i.e., how far ahead the agent considers when making decisions, in the range (0,1]. The larger the γ is, the more steps the agent considers going forward, but the more difficult it is to train; the smaller the γ is, the more the agent focuses on immediate benefits and the easier it is to train. Although we want the agent to think in the long term, excessively high discount factors may impede algorithm convergence. As observed in Figure 10, training proceeded swiftly when γ=0.4, and the best result was achieved when γ=0.9. On the other hand, the algorithm trained most slowly when γ=0.99, implying that, while unfinished services can influence dynamic deployment, it is not necessary to consider overly long-term impacts in a limited training cycle.

**Figure 11:**
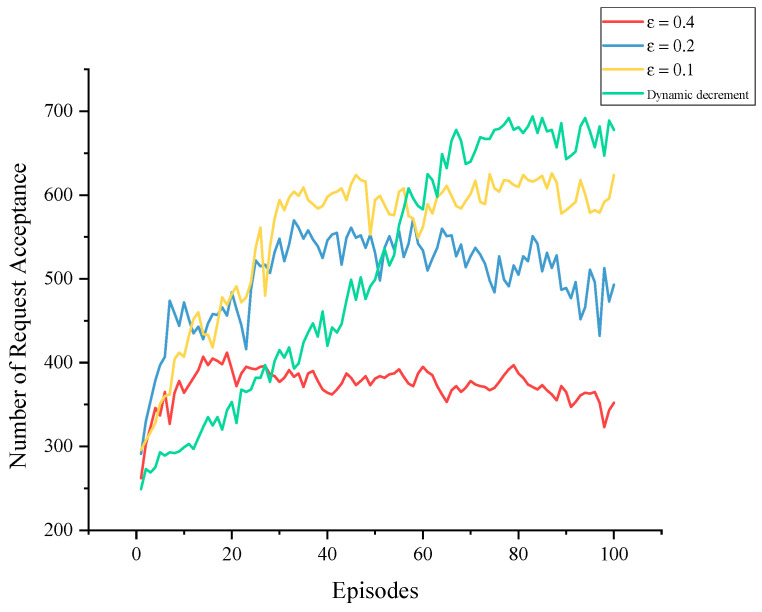
Training processes with different ε values.

The training used the ε−greedy approach to enhance the agent’s exploration ability. ε represents the probability that the agent will randomly choose an action. The best states are often explored only with a good enough Q-network. If ε is too large, excessive exploration in the later stages of training may affect the utilization of acquired knowledge and hinder further performance improvement. If ε is too small, on the other hand, it may lack sufficient exploration in the early stages. Instead of setting ε to a fixed value, the dynamic decrement strategy started with ε=0.9, the strongest exploration, and gradually reduced it in a linear fashion until it reached 0.05, and then it remained constant. This approach provided adequate exploration in the initial stages and maximized the use of acquired knowledge in the later stages. The simulation results depicted in Figure 11 demonstrate that the dynamic decrement strategy prolonged effective learning time and ultimately yielded better training outcomes.

**Figure 12:**
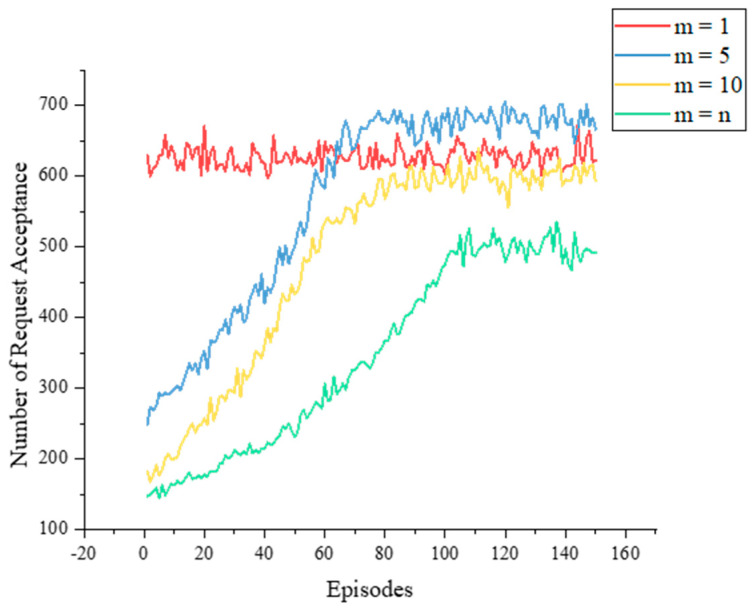
Training processes with different m values.

The impact of varying m on agent training is shown in Figure 12. The MDQR algorithm requires an appropriate value of m to balance between exploration and exploitation. When m is too small, it approaches the LBSP algorithm (m = 1), which limits the selection space and may miss better actions. When m is too large, it approaches the original DQN algorithm (m = n), which increases the selection space and may cause training difficulty. Therefore, choosing a suitable value of m is crucial for the performance of the MDQN algorithm. In our simulation experiments, we achieved the best results when m was 5.

## 6. Conclusions

This paper focuses on the problem of how to deploy dynamically arriving SFC requests more effectively on the SDN/NFV-enabled network. We propose a DRL-based method MQDR that reduces the action space of the intelligent agents to facilitate their training and enhance their performance. We also demonstrate that performing some readjustment on the basis of the SFC request information and the underlying network state before deploying the SFCs can improve the load balancing of the network and increase its capacity to accommodate more SFCs. Simulation experiments indicate that our approach is suitable for online deployment in dynamic networks; it increases the request acceptance rate by approximately 25% compared with the original DQN algorithm and by 9.3% compared with the Load Balancing Shortest Path (LBSP) algorithm. However, despite considering dynamic SFC request arrival, our study assumes that the SFC traffic remains constant following the request arrival. In future work, we will delve more deeply into deploying and readjusting service chains when both SFC request arrival and traffic are dynamic.

## Figures and Tables

**Table 1:** Notations used in the model.

Notations	Description
G	The underlying network
$V^{S}$	Set of underlying nodes, $v_{i}^{s}\in V^{s}$
$E^{S}$	Set of underlying links, $e_{ij}^{s}\in E^{S}$
$e_{ij}^{s}$	Underlying link between $v_{{}_{i}}^{s}$ and node $v_{{}_{j}}^{s}$
$BW_{ij}^{S}$	Bandwidth of $e_{ij}^{s}$
$L_{ij}^{S}$	Link latency of $e_{ij}^{s}$
F	Set of VNF types, f∈F
$coeff_{c}^{f}$	CPU resource factor of VNF type f
$\theta^{f}$	Traffic scaling factor of VNF type f
R	Set of SFC requests, $r^{j}\in R$
$V_{j}^{NF}$	Set of ordered NF requests, $v_{j,u}^{nf}\in V_{j}^{NF}$ denotes the u-th NF
$E_{j}^{NF}$	Set of logical links of $r^{j}$ $,\;e_{j,uv}^{nf}\in E_{j}^{NF}$
$c_{j,u}^{nf}$	CPU resource requirement of $v_{j,u}^{nf}$
$bw_{j,uv}^{nf}$	Bandwidth requirement of logical link $e_{j,uv}^{nf}$
$l_{j}$	End-to-end delay allowed for the request $r^{j}$

**Table 2:** Decision variables.

Notation	Description
$p_{j}$	To show whether $r^{j}$ has been successfully deployed.
$t_{j,u}^{f}$	To show whether $v_{j,u}^{nf}$ is of type f.
$\eta_{j,u}^{i,t}$	To show whether the u-th virtual node $v_{j,u}^{nf}$ is deployed on physical node $v_{i}^{s}$ when t.
$\tau_{j,uv}^{pq}$	To show whether traffic segment $e_{j,uv}^{nf}$ flows through physical link $e_{pq}^{s}$
$\beta_{f}^{i}$	To show whether there is a VNF of type f on physical node $v_{i}^{s}$.

**Table 3:** Performance of DQN with different numbers of hidden layers.

Number of Hidden Layers	Request Acceptance Rate
2	0.56
3	0.71
4	0.68

## Data Availability

Data sharing is not applicable to this article.
